# Integrated computational and experimental pipeline for quantifying local cell–matrix interactions

**DOI:** 10.1038/s41598-021-95935-2

**Published:** 2021-08-12

**Authors:** Hugh Xiao, Ryan Y. Nguyen, Ryan LaRanger, Erica L. Herzog, Michael Mak

**Affiliations:** 1 Department of Biomedical Engineering, Yale University, New Haven, CT USA; 2 Department of Medicine (Pulmonary, Critical Care and Sleep), Yale University School of Medicine, New Haven, CT USA

**Keywords:** Respiratory tract diseases, Imaging

## Abstract

Cellular interactions with the extracellular matrix (ECM) play a key role in modulating biological processes. While studies have identified key molecular factors of these interactions, the mechanical regulation associated with these interactions is not well characterized. To address this, we present an image analysis platform to analyze time-dependent dynamics observed in lung fibroblasts embedded in a 3D collagen matrix. Combining drug studies with quantitative analysis of cell–matrix interactions, our results are able to provide cellular level quantitative insights for mechanical and biophysical phenomena relevant to cell-ECM interactions. This system overall represents an initial pipeline for understanding cell mechanics in a 3D collagen gel and their implications in a physiologically relevant context.

## Introduction

Cellular interactions with the extracellular matrix (ECM) encompass a broad range of biophysical phenomena which are necessary for a variety of biological processes such as cell anchoring, migration, proliferation, and differentiation^1,2^. Cells are able to mechanically interact with the ECM and translate these interactions into subsequent signaling events which, in turn, drive further cellular interactions with the surrounding stroma^3^. The interplay of these events is crucial for normal cellular functions, and its dysregulation has been associated with diseases such as lung fibrosis and cancer^4^. To accurately study these phenomena, highly quantitative in vitro systems which capture these mechanobiological interactions must be developed.

3D biomimetic hydrogels have been used to study a variety of biological phenomena, including tissue development, stem cell differentiation, and cancer metastasis^5–8^. Within these gels, cells have the capability of physically interacting with and remodeling the matrix itself in a variety of ways. Driven by cellular cytoskeletal mechanics, cells can induce tensional forces on the matrix which can cause matrix densification and alignment^9^. Such remodeling has been associated with increased cell migration and worse prognosis in disease, including fibrosis^10–12^. In addition to physical cues, cells may also molecularly interact with the matrix via matrix metalloproteinases (MMPs) which degrade the matrix. In particular, MMPs have been implicated in lung fibrosis as their expression has been linked to profibrotic activity, inflammation, and altered immune function^13–15^. Together, these physical and biochemical interactions facilitate cell–matrix interactions.

Due to the complex nature of these molecular and physical interactions, quantitative metrics to study how cells interact with the matrix have been developed to derive measurements of cell behavior in the context of 3D matrix remodeling^16–20^. While these metrics extract useful information from these in vitro datasets, their corresponding outputs mostly focus on 3D invasion dynamics of cells. However, these studies spend less effort interrogating the local cell–matrix interactions which underlie these dynamic phenotypes. In addition, studies which assess these biophysical phenomena focus on bulk gel deformation and do no not focus on local cell–matrix interactions^21^. Thus, there is a need for a pipeline to quantify these 3D biophysical cellular responses.

To address this, we present a scalable in vitro assay and quantification which combines 3D culture in collagen and live-cell imaging. We apply our system to idiopathic pulmonary fibrosis (IPF) and normal human lung fibroblast (NHLF) cell lines. These cultures can be made in a multi-well plate format and demonstrate differences in remodeling. These 3D gels can be cultured and imaged over many days to observe the mechanobiology of cell–matrix interactions. The fibrotic cell spheroids are dyed with CellTracker Green CDMFA and are seeded in 3D collagen gels. To produce high resolution images of the individual collagen fibers in relation to the fibroblasts, we mix fluorescent polystyrene microspheres to the collagen as has been done previously to produce high quality images of collagen fiber movement^22^. To assess the collagen fiber movement, we quantify three metrics: spheroid-collagen pulling velocity, collagen density around the spheroids, and spheroid hole formation. These programs are able to track spheroid-matrix interactions in 3D collagen gels and determine parameters such as rate of collagen densification and hole formation in gels. Our metrics highlight cell–matrix interactions including force and MMP-mediated events and allows for interrogation of biophysical interactions in 3D microenvironments.

## Materials and methods

### Cell culture

Primary cell lines used in this study were a gift from Dr. Erica L. Herzog (Yale). NHLF29800 (batch# 548315), NHLF29319 (batch# 511473), and NHLF29729 (batch# 543644) are normal human lung fibroblasts from Lonza (cat.# CC-2512). IPF29548 (batch# 6F5002) is diseased human lung fibroblast from Lonza (cat.# CC7231). IPF17992 (batch# 216647) and IPF16769 (batch# 219666) are diseased human lung fibroblasts from ASTERAND/BIOSCIENCE (cat.# PCR-70-0214). The numbers following the letters—29800, 29319, 29729, 29548, 17992, 16769—are tissue acquisition numbers (Lonza) and donor IDs (ASTERAND/BIOSCIENCE), which correlate with individual patients. We use the combination of cell line types (NHLF or IPF) and tissue acquisition number/donor ID throughout this paper to distinguish the cell lines.

The cell lines were cultured in Dulbecco's modified Eagle's medium (DMEM, ThermoFisher, cat.# 11965092), with 10% fetal bovine serum (FBS) (ThermoFisher, cat.# 16000-044) and 1% pen/strep (Life Technologies, cat.# 15140122). Cells were cultured at 37 °C at 5% CO_2_ with media changed every 2 days and passaged at 70–80% confluence.

### Confocal microscopy

A Leica SP8 confocal microscope (Wetzlar, Germany) with a 10× objective was used to image spheroids. A temperature of 37 °C and a 5% CO_2_ atmosphere were maintained using a humidified live-cell imaging incubator (OKOlab). For each gel, a ~ 250 µm-thick *z-*stack was imaged with a 5 μm z-step size. We performed time-lapse microscopy over the first 12 hours and single-time-point snapshots on day 5.

### Spheroid formation

To overcome single cell variability and to simulate a more tissue-like organization of cells^23^, we made fibroblast spheroids to measure their remodeling of the collagen matrix. Spheroids were composed of either IPF or NHLF cells. They were prepared based on established protocols^24^. In brief, we prepared 50 μL agarose gels (Sigma Aldrich, cat.# A9539-500G) in each well of a 96-well polystyrene cell culture plate (Greiner Bio-One, cat.# 655180). To visualize the spheroids, before forming the cell aggregates, we dyed the cells with CellTracker Green CDMFA (10 μM, ThermoFisher Scientific, cat.# C2925). We then deposited 1000 fluorescently labelled cells to each well so that each cell aggregate on day 0 has 1000 cells. The plate was then centrifuged at 20 °C, 400 rcf for 10 min. The cells were then incubated at 37 °C with 5% CO_2_ from day 0 to day 4. Cells aggregated into spheroids during the time. On day 4, the spheroids were picked up using a wide-tip pipette (Corning, cat.# T-205-WB-C-S) and were embedded in collagen gels.

### Collagen gel preparation and encapsulation

We embedded our spheroids in collagen gels to mimic the 3D physical environment of the lung extracellular matrix. The collagen gels were made by adding a titrated amount of 0.5 N NaOH to neutralize a mixture containing double-distilled H_2_O, 10× PBS (containing phenol red) diluted 1:10 to the final volume of the collagen solution, and acetic acid solubilized type I rat tail collagen (Corning, cat.# 354249). The solution was prepared on ice for a final collagen concentration of 2 mg/mL. To track collagen movement, we added carboxylated polystyrene fluorescent microspheres (ThermoFisher Scientific, cat.# F8807) to the mixture. We also added the treatment to the mixture to expose the spheroids to the treatments before collagen polymerization starts. The treatment and their concentrations are 10 ng/mL TGF-β (PeproTech, cat.# 100-21), 5 μM Nintedanib (Santa Cruz, cat.# 656247-18-6), 20 μM GM6001 (abcam, cat.# ab120845), or 100 μM SMIFH2 (abcam, cat.# ab218296). 50 μl of the collagen gel mixture was then pipetted into each well of a 24-well glass-bottomed cell culture plate (MatTek, cat.# P24G-0-10-F) kept on an ice pack. Once all gels were transferred to the 24-well plate, we transferred the plate to an incubator, and the collagen was allowed to polymerize for 1 hour at 37 °C with 5% CO_2_. At the beginning of the gelation, the plate was flipped several times to prevent spheroid sedimentation to the bottom of the plate. After 1 hour, fresh media with appropriate treatments were added to each well and were subsequently maintained at 37 °C with 5% CO_2_. Drug media were replaced every other day.

Prior to depositing the collagen, plates used for collagen gel seeding were surface-coated with polydopamine (Sigma Aldrich, cat.# H8502-10G). We incubated plate surfaces for 2 hours with 0.5 mg/mL polydopamine dissolved in 10 mM, pH 8.5 Tris/HCl buffer (bioWORLD, cat.# 42024014-1) before washing with 1× PBS three times. This allows for collagen gels to adhere to the surface of the plate and prevents detachment of the gels^25^.

To observe cell viability on day 5, we incubated the sample with SYTOX Green nucleic acid stain (diluted to 100 nM, Thermo Fisher Scientific, cat. # S7020), which is a dead cell indicator, and 4′,6-diamidino-2-phenylindole (DAPI, diluted to 2 drops/mL, Thermo Fisher Scientific, cat.# R37610) for 15 minutes at 37 °C. After the incubation, the sample was washed with 1× PBS before imaging.

To observe the protein distributions on day 5, after fixing the samples with 4% paraformaldehyde (Santa Cruz Biotechnology, cat.# 30525-89-4), we incubated the sample with Alexa Fluor 633 NHS Succinimidyl Ester (100 μg/mL in 0.1 M NaHCO_3_, Thermo Fisher Scientific, cat. # A20005) at room temperature for 1 hour. After thorough washing with 1× PBS, the sample was incubated with Hoechst 33342 (5 μg/mL, Thermo Fisher Scientific, cat.# H3570) and Alexa Fluor 488 Phalloidin (1 unit/mL which is approximately 33 nM, Thermo Fisher Scientific, cat.# A12379) for 15 minutes at 37 °C. After the incubation, the sample was washed with 1× PBS before imaging.

### Statistical analysis

To study the treatment effects, we compared across conditions. Because we observed collagen densification in both control and treatment groups, we compared the hour 6 densification of each spheroid to its densification at hour 0 for each condition to find the hour 6 collagen density fold change. In these comparisons, we used two-tailed, one-sample Wilcoxon signed rank tests with Benjamini–Hochberg correction with a false discovery rate of 5%. The significance is assigned accordingly. Because each data point of collagen density at hour 6 was normalized by collagen density at hour 0, we compared each data point to a collagen density fold change of 1 (which is indicative of no change in collagen density between the two time points) for the Wilcoxon test (Figs. 4B-C, S7 A-B, S8A-B and E-F).

To compare across the conditions, statistical analyses of holes were conducted with a one-way Kruskal Wallis test followed by Dunn’s post-hoc comparison test (Figs. 4D, S7C-D, S8C-D and G-H).

To further account for inter-patient heterogeneity, for each cell line, we also normalize the above metrics by the mean of the TGF-β (only) treated group of the corresponding line (Figs. 3B–D, 4B–D, S6, and S7).

The error bars in the graphs are the standard error of the mean (s.e.m.). Values with *P* < 0.05 were considered statistically significant. The * symbol indicated *P* < 0.05, and ** was for *P* < 0.01. When there was no significant difference, no * symbol was assigned.

### Ethics approval

Ethics approval is not required for this study.

## Analysis algorithms

### Overview

**Figure 1:**
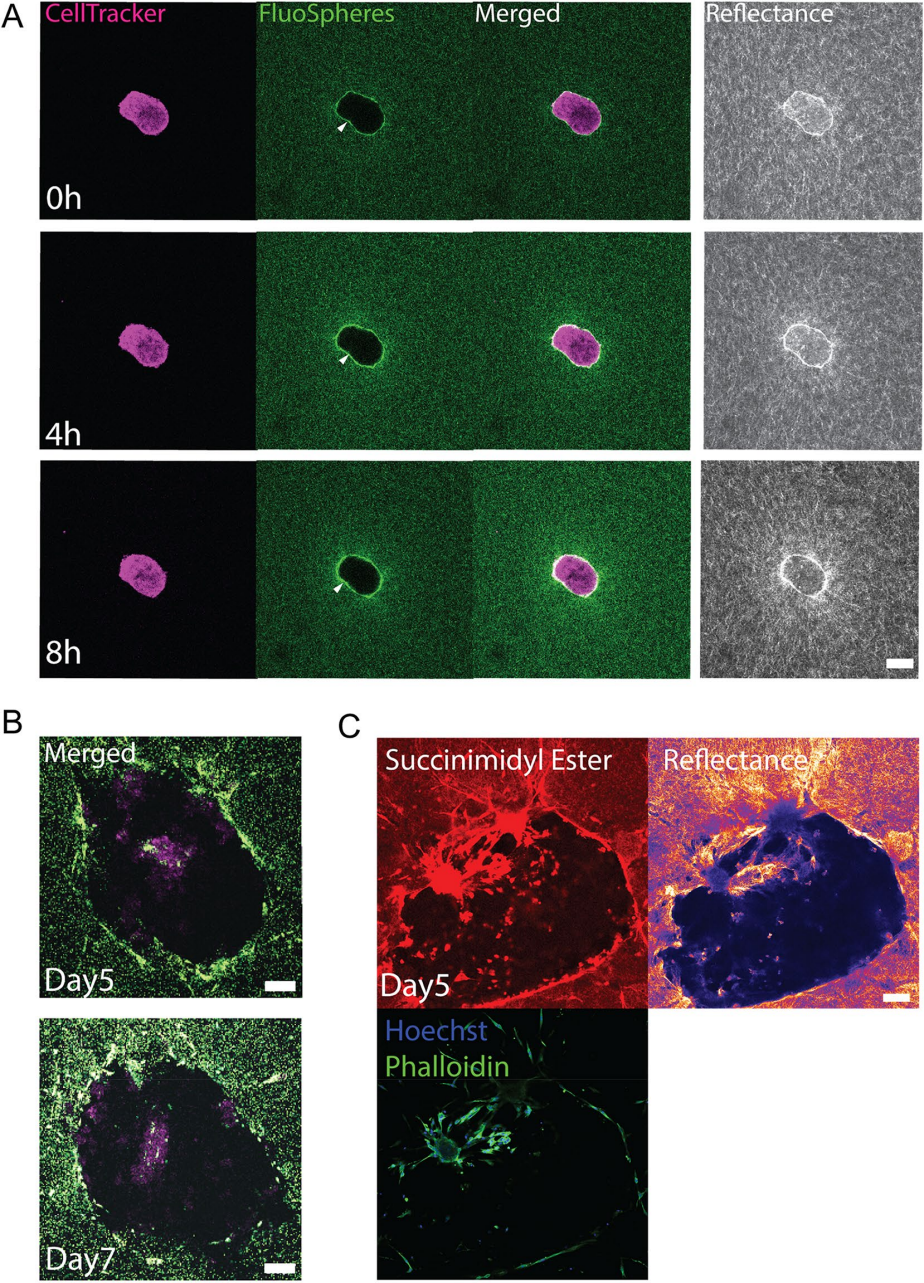
IPF spheroids interact with collagen gels. (**A**) Time series of an IPF spheroid (magenta) treated with TGF-β pulling collagen (green) at 0, 4, and 8 hours after being cultured in collagen gels show collagen densification around the spheroid. (From left to right) Images show spheroid labeled with CellTracker dye, fluorescent microbeads mixed in the collagen gel, merged, and reflectance images taken by confocal reflectance microscopy. (**B**) Representative fluorescence images of an IPF spheroid that is treated with TGF-β on day 5 and day 7. Degradation and rupture of collagen matrix lead to hole formation. (**C**) Representative fluorescence images of an IPF spheroid that has been treated with TGF-β showing that no amine groups (the dark regions that fail to be dyed with fluorescent succinimidyl ester) or visible structures (the dark region in the reflectance image) in the hole on day 5. Scale bars, 100 μm.

We present two image analysis platforms of 2D and 3D analyses to study spheroid behaviors—the 2D analyses are performed on 2D image data (in-focus images that are manually selected), and 3D analyses are performed on 3D z-stack image data. Spheroids chosen for analyses are manually checked to ensure that they are not initially too close to the bottom of the dish and are fully encapsulated by collagen. Spheroids are processed according to the particular timescale dynamics we want to observe. For imaging taking place over the first 12 hours after spheroids are seeded into the gel, we observe densification of collagen around the spheroids (Fig. 1A, white arrows). To quantify such densification, we investigate (a) the collagen-pulling rate as measured by the speed of the microbeads, (b) collagen densification fold change as measured by pixel brightness, and (c) persistence distance of the remodeling as measured by the pixel brightness over distance. In later days, spheroids cause hole formation in the collagen gels (Fig. 1B). As such, we measure (d) the volumes of these holes (Fig. S8H) and their area at the z-slice with the maximum hole area (Fig. S8G). Of note, most cells are alive on day 5 (Fig. S9), and that the holes are voids rather than secreted extracellular proteins (Fig. 1C). The programs are implemented in MATLAB 2020b^26^ and Python 3.6^27^. The scripts are available upon request.

#### Collagen-pulling is measured as microbead speed

**Figure 2:**
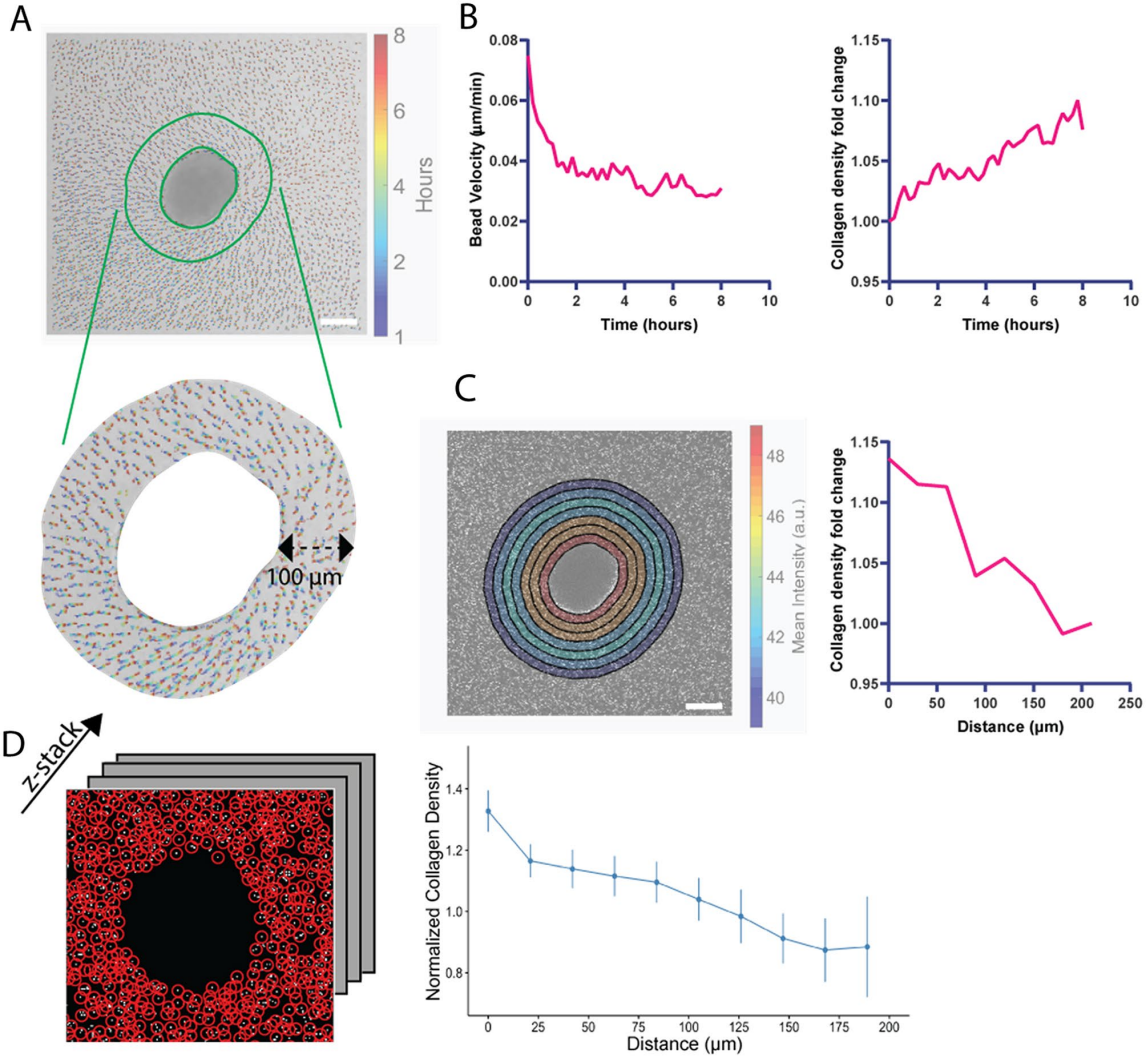
Analytical metrics: bead velocity and collagen density. (**A**) Example image of bead trajectory tracking using the Blair and Dufresne routine for an IPF spheroid with the color indicating time. The cut-out ring (bottom) is where mean intensity or bead velocities are averaged. Scale bar: 100 μm. (**B**) Mean velocity of the beads decreases over time (left), and collagen density increases over time (right). (**C**) Collagen density decreases with an increasing distance away from the spheroid, shown in the heatmap (left) and its profile plot (right). The analyzed image is a spheroid cultured in collagen for 6 h. (**D**) Example image of collagen bead tracking via TrackPy. Dark region in the middle is a spheroid. Its profile plot is on the right.

To understand collagen-pulling, we quantify the speed of the movement of encapsulated fluorescent polystyrene microspheres in collagen. To track beads, we used the open-sourced Blair and Dufresne routine^28^, which has been implemented in MATLAB. Bead tracking confirms a general collagen movement towards the spheroid (Fig. 2A). We calculate the averaged bead speed in a ring that is 0–100 µm around the spheroid, which we determine as a characteristic distance for bead-pulling (Figs. S2C and S5C). This method is able to capture a decrease in bead-pulling over time (Fig. 2B left, Figs. S2A, and S5A).

#### Collagen densification is measured as the brightness change

To understand the densifying collagen around spheroids, we quantify the brightness change as a result of the crowding beads around the spheroid in both 2D timelapse images and 3D z-stacks.

##### 2D timelapse images

**Figure 3:**
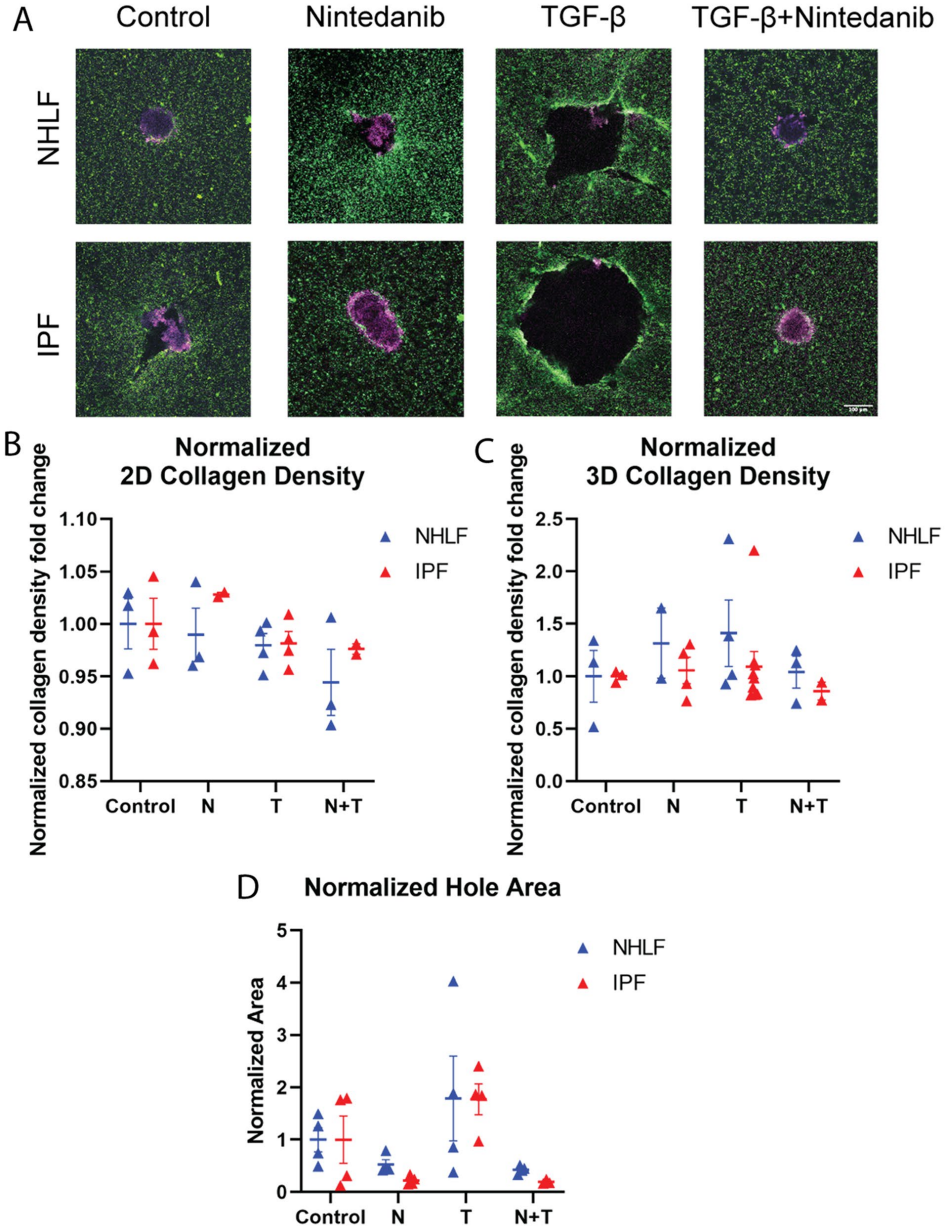
Analyses of TGF-β and Nintedanib-treated spheroids. (**A**) Day 5 NHLF29800 and IPF29548 spheroids treated with Nintedanib, TGF-β, TGF-β + Nintedanib. Scale bar, 100 μm. (**B**) 2D collagen density fold change at hour 6 normalized by the TGF-β only condition for NHLF29800 and IPF29548 spheroids (each data point is a spheroid). (**C**) 3D collagen density fold change at hour 6 normalized by the TGF-β only condition for NHLF29800 and IPF29548 spheroids (each data point is a spheroid). (**D**) Day 7 hole size normalized by the TGF-β only condition for NHLF29800 and IPF29548 spheroids in different conditions (each data point is a spheroid). T stands for TGF-β. N stands for Nintedanib.

**Figure 4:**
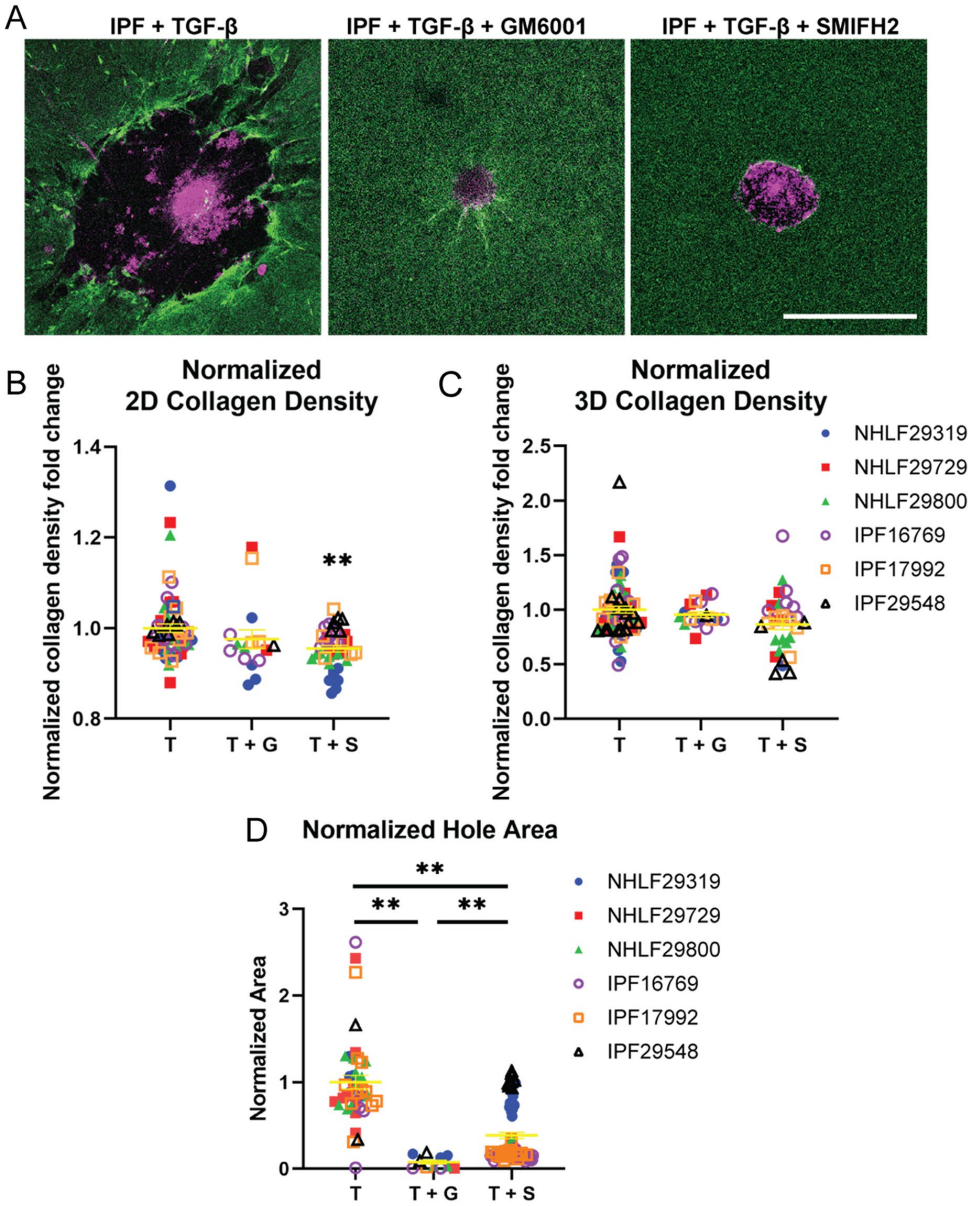
Analyses of TGF-β, GM6001 + TGF-β, and SMIFH2 + TGF-β treated spheroids. (**A**) Day 5 IPF spheroids treated with TGF-β, GM6001 + TGF-β, and SMIFH2 + TGF-β. Scale bar, 500 μm. (**B**) 2D collagen density fold change at hour 6 after seeding normalized to each spheroid’s hour 0 collagen density and by the TGF-β only condition for each cell line for IPF and NHLF spheroids. Significance is assigned based on one-sample, two-tailed Wilcoxon ranked sign test with Benjamini–Hochberg correction. (**C**) 3D collagen density fold change at hour 6 after seeding normalized to each spheroid’s hour 0 collagen density and by the TGF-β only condition for each cell line for IPF and NHLF spheroids. Significance is assigned based on one-sample, two-tailed Wilcoxon ranked sign test with Benjamini–Hochberg correction. (**D**) Day 5 hole size normalized by TGF-β only condition for each cell line for NHLF and IPF spheroids in different conditions. Significance for hole size differences is assigned based on one-way Kruskal Wallis test followed by Dunn post-hoc comparison tests. T stands for TGF-β. G stands for GM6001. S stands for SMIFH2.

We calculate the averaged bead intensity (measured by the average pixel intensity) in a ring of 100 µm around the spheroid, and this is the same ring used for averaging bead velocities (Fig. 2A). To normalize for a baseline collagen density, we normalize the brightness by that at hour 0. Spheroid periphery is traced out using Active Contour^29^ (an available function in MATLAB). Our results show that collagen density increases over time (Figs. 2B right,  S2B, and S5B). During our drug studies, we further normalize our collagen density fold change to the mean of our control group (Figs. 3B, 4B, S6A, and S7A).

##### 3D z-stacks

Because collagen-pulling by an encapsulated spheroid happens in three dimensions, we further quantify the collagen density change around a spheroid in its z-stack (Fig. 2D). To measure the average bead density around the spheroid, we use TrackPy^30^ to locate the beads, calculate the average intensity of these beads within a spherical shell near the spheroid, and divide the sum intensity by the volume of the shell (Fig. S1B). The spherical shell is centered at the spheroid’s centroid. This centroid is determined by tracing out the spheroid at each z-slice using Canny Edge detection^31^ and then calculating the centroid location with this reconstructed 3D spheroid (Fig. S1B). Because quantifying 3D data is time-consuming and computation-intensive, this analysis was applied to single-time points rather than a timelapse. In our subsequent analyses, this method is used to measure hour 6 collagen density around the spheroid and compare across treatments (Figs. 3C, 4C, S6B, S7B, S8B, and S8F). We use the collagen density 25 μm away from the spheroid edge and calculate fold change of hour 6 compared to hour 0 after being cultured in collagen. The spheroid edge is manually determined by finding a significant spike in 3D collagen density data. We use the distance between the spike and the centroid to estimate the spheroid radius (*r* as seen in Fig. S1B). A detailed discussion comparing the two segmentation tools—Active Contour and Canny Edge detection—can be found in Supplementary Note 1. An extended description and subsequent validation of the 3D collagen density algorithm can be found in Supplementary Note 2. During our drug studies, we further normalize our collagen density fold change to the mean of our control group (Figs. 3C, 4C, S6B, and S7B).

#### Persistence distance of the pulling is measured as brightness gradient

To understand the persistence distance of the collagen-pulling by the spheroids, we quantify microbead brightness as a function of distance from spheroids. We calculate the averaged bead intensity (measured by the average pixel intensity) in concentric rings around the spheroid, each with a width of 30 μm (Fig. 2C left). 30 μm is an arbitrary choice that is not too narrow as to be mainly affected by noises but also narrow enough to capture a gradual decrease in the collagen density away from the spheroid periphery. To correct for the brightness of different images and the variation of baseline brightness of the microbeads, the data is normalized by dividing the average mean intensity of each ring by that of the outermost ring (210–240 μm). Our results show that collagen density decreases with an increasing distance away from the spheroid (Figs. 2C, S2C, and S5C).

#### Hole volume is measured in 3D z-stacks, and its 2D hole size is represented by its area at the z-slice with the maximum hole area

To understand the treatment effects on collagen hole formation, we manually trace out the hole sizes in Fiji^32^. The image to trace is picked out by finding the z-slice that displays the maximum hole size. To cross-validate the results, we also quantify the 3D hole volume using a custom Python script that uses Canny Edge detection^31^ to trace out the holes in each z-slice and then sums their area multiplied by the z-step size for the total volume of the hole (Figs. S1A and S8H). During our drug studies, we further normalize our hole measurements to the mean of our control group (Figs. 3D, 4C, S6C-D, and S7C-D).

For the above analyses, manual verification is conducted. We use visual validations to identify erroneous characterization by the automated analysis. The misidentified events were excluded from the final plots.

## Results

### NHLF and IPF spheroids contract collagen and produce holes

To validate our system and the ensuing analytical metrics, we seeded normal human lung fibroblast (NHLF) and diseased lung fibroblast (IPF) spheroids in fluorescent collagen with and without Nintedanib and with and without 10 ng/mL TGF-β (Fig. 3A). We perform time-lapse microscopy over the first 12 hours and take single-time-point snapshots on day 5 (Fig. 3A). We choose Nintedanib because it is a current clinical option for treating IPF^33^. We hypothesize that Nintedanib would abrogate collagen densification by the fibroblasts.

To understand the pulling dynamics over the course of the first several hours after seeding, we quantify time-dependent spheroid interactions with the matrix by calculating 2D bead velocity (Fig. S2A), 2D collagen density fold change over time (Fig. S2B), and 2D collagen density fold change over distance (Fig. S2C). All three metrics are able to capture the process of collagen densification. There is a consistent bead-pulling by the spheroids over time, and their speeds gradually decrease (Fig. S2A). Consistent with the collagen-pulling, collagen density near the spheroid increases over time (Fig. S2B). We find no significant correlative changes in collagen density after 6 hours across all treatments (Figs. 3B–C).

The spheroids further remodel the matrix over 5 days, and in the TGF-β stimulated spheroids, holes form in the matrix near the spheroids for both NHLF and IPF spheroids. We use the manual 2D hole traces to capture hole formation (Figs. 3D and S6C).

In summary, we initially hypothesized that collagen velocity would be important for disease progression, as increased ECM concentration is a hallmark of fibrotic disease. However, based on our experimental setup and measurements, although we are able to capture increases in the collagen density over time near the spheroids, these increases do not appear to be robust metrics to distinguish between the drug conditions or between IPF and NHLF. In addition, we identify another matrix-remodeling signature–hole formation.

### GM6001 and SMIFH2 inhibit the hole formation

We next aim to determine the role that anti-remodeling drugs might have on fibroblast activity. We tested both GM6001 and SMIFH2. GM6001 is a pan-inhibitor of matrix metalloproteinases (MMPs)^34,35^. SMIFH2 is an inhibitor of actin nucleation, targeting the formins^36^.We choose to study GM6001 because cells express MMPs to degrade collagen ^37^. We choose to study SMIFH2 because the cytoskeleton is a key regulator of cell–matrix interactions^38^. To observe cell-line specific mechanobiological heterogeneity, we culture primary human lung fibroblast spheroids from three NHLF and three IPF cell lines in 3D collagen gels. These spheroids are cultured in bead-labeled collagen gels over 5 days with all of the spheroids incubated in TGF-β (Fig. 4A).

To understand how different metrics are affected by the treatments, we quantify collagen density changes and hole formation. We observe collagen densification in both the control and treatment conditions and in both the NHLF and IPF cell lines (Figs. S3 and S4). We also observe heterogeneity in response to drug treatments across cell lines (Figs. S3, S4, and S8). Although we hypothesize that these drugs would severely reduce collagen remodeling, GM6001 and SMIFH2 treated conditions still show initial collagen recruitment (Figs. 4B-C, S7A-B, S8A-B,E-F). This could be due to the drugs being added after gelation, and that it takes some time for the drugs to take effect. When analyzing collagen density, the different cell lines behave similarly for each condition (Figs. S8E-F). Therefore, we group spheroids from all cell lines together for each drug condition (control, GM6001, and SMIFH2) in Figs. 4B-C.

To understand how the treatments affect hole formation, we compared the hole areas and volumes on day 5 across treatments (Figs. 4D, S7C-D, S8G-H). The absence of large hole formation under TGF-β treatment may be explained by cell line heterogeneity (and thus inherent resistance to these drugs) or by the induction of drug treatment after gelation. Our data suggests that the presence of GM6001 or SMIFH2 appears to reduce hole formation.

Overall, our results show that neither GM6001 nor SMIFH2 have drastic influences on ECM accumulation but may be effective in reducing hole formation. The absence in ECM accumulation may likely in part be due to the experimental setup in which the drugs are added after collagen gelation, and additional time may be needed for the drugs to take effect before impacting ECM accumulation.

## Discussion

Creating an experimental-quantitative system to study the cellular interactions with the ECM has the potential to reveal molecular insights into the dynamic phenotypes which underlie biological processes and disease. We embed primary fibroblasts into collagen hydrogels to study these phenotypes. Using a system which can take cells isolated from patients to evaluate their behaviors in a 3D collagen system may allow us to parse the direct impact of cellular and biophysical elements on cell–matrix interactions, such as actin cytoskeletal forces, matrix metalloproteinase activity, and heterogeneity of patients displaying fibrotic diseases. Our system has the potential to be used for assessing dynamic mechanobiological phenomena in a physiologically relevant context.

Our experimental system also shows collagen densification as a method of matrix remodeling by lung fibroblasts. We hypothesized that collagen velocity or densification would be important for disease progression, as increased ECM concentration is a hallmark of fibrotic disease. However, our quantification is unable to robustly detect differences between the normal and diseased cell lines or between control and treatments. This could be due to the drugs being added after gelation, and that it takes some time for the drugs to take effect. In future studies, this can be addressed by a pretreatment of the spheroids in the drugs before being encapsulated in the matrix.

An important consideration to this study is cell line variability between and among healthy and diseased cell lines. For example, in our experimental system, only 1 of the 3 IPF cell lines (IPF17992) is able to consistently form holes in our study (Fig. S8G-H), though this physical tearing of the matrix is reminiscent of honeycombing in IPF patients^39^. However, 2 of the 3 NHLF cell lines (NHLF29800 and NHLF29729) are also able to form holes (Fig. S8G-H). Such observed heterogeneity makes classification of clinically relevant phenotypes challenging. It is known that NHLF and IPF cell lines are heterogeneous, and this heterogeneity may explain differences in mechanical responses^40^. Furthermore, the clinical definition of honeycombing also requires molecular profiling including the presence of mucus as well as the loss of type II alveolar epithelial cells^39^. This molecular profiling may be able to further differentiate between NHLF and IPF cell lines. In our future studies, we hope to associate these molecular signatures with the mechanical tearing observed in our in vitro system.

Future studies will also look at other prominent mechanical phenotypes such as the local alignment of collagen fibers around cells. Fiber alignment is known to impact cell behaviours^41^, and it is a useful metric to quantify collagen remodeling by cells^42^. In addition, previous research has suggested that diseased fibroblasts are able to stiffen the ECM in vitro which implicates physical parameters of the collagen gel as variables of disease progression^5^. Tuning physical parameters of the collagen gel, such as stiffness via addition of chemical crosslinkers and varying concentration, will allow for a hydrogel system that better mimics the tissue microenvironment. Another aspect that needs to be explored when making claims about the molecular targets and their roles in disease progression is the effect of various dosages on altering cell behaviour. SMIFH2, for example, has been shown to affect cell viability at high concentrations^43^. Ultimately, detailed characterization of local dynamic phenotypes in more physiologically relevant contexts will reveal further insights into the role of cell–matrix interactions in biological processes and diseases.

## Conclusions

Cell–matrix interactions are complex phenomena with spatiotemporally dependent biophysical phenotypes. Underlying drivers are not fully understood, and model systems and analytic approaches are limited toward studying these phenotypes. Here, we present an in vitro model that can be scaled in a multi-well plate format that can capture certain biophysical features, notably ECM densification and hole formation, in some fibroblast cell lines. We further develop integrated computational tools that can extract quantitative information from these observations. Our findings highlight a dynamic ECM landscape with multiple sequential stages. Our integrated experimental and computational pipeline provides a systematic approach for future investigations on emergent biophysical features relevant to cell matrix interactions.

## Supplementary Information

Supplementary Information.

## Data Availability

The data that support the findings of this study are available from the corresponding author upon reasonable request.

## Code availability

The custom Python and MATLAB analysis codes are available from the corresponding author upon reasonable request.
