# Supplementary material for: Integrated computational and experimental pipeline for quantifying local cell–matrix interactions
Source: Sci Rep. 2021 Aug 12;11:16465. doi: 10.1038/s41598-021-95935-2 (PMC8361134; doi:10.1038/s41598-021-95935-2)
Supplement: Supplementary file 1 — Supplementary Information. [file 41598_2021_95935_MOESM1_ESM.docx]

**Supplementary Information**

**Integrated Computational and Experimental Pipeline for Quantifying Local Cell-Matrix Interactions**

**Hugh Xiao^1^*****, Ryan Y. Nguyen^1^*****, Ryan LaRanger^1^, Erica L. Herzog^2^, Michael Mak^1^**

**Affiliations**

^1^ Department of Biomedical Engineering, Yale University, New Haven, CT, USA

^2^ Department of Medicine (Pulmonary, Critical Care and Sleep), Yale University School of Medicine, New Haven, CT, USA.

* These authors contributed equally

Correspondence and request for materials should be addressed to M.M. (michael.mak@yale.edu)

**Supplementary Text**

**Supplementary Note 1: A comparison of the segmentation tools: Canny Edge detection vs. Active Contour**

We use Active Contour[^1^](https://paperpile.com/c/z5cTKl/Xicy8) to trace the contour of in-focus spheroids and use Canny Edge detection[^2^](https://paperpile.com/c/z5cTKl/WEDm0) to trace spheroid contour in each slice of z-stacks. While Active Contour is better at tracing boundaries of an in-focus object in the brightfield, Canny Edge detection is better at tracing the boundaries of dark regions in fluorescent images. When going through z-stacks produced by confocal microscopy, Active Contour has trouble identifying the boundaries of out-of-focus spheroids in the brightfield. On the other hand, Canny Edge detection can trace the spheroid boundary by tracing the dark void in the fluorescent channel (an example of a spheroid surrounded by fluorescent microbeads can be found in the second column of Fig. 1A), and thus Canny Edge detection is better at tracing the boundaries of a spheroid in a z-stack which would contain out-of-focus brightfield images. For the above reasons, we use Canny Edge detection to trace the collagen holes in 3D hole volume analysis rather than using Active Contour. See Fig. S9 top row and Fig. 1B for example images of the hole in the brightfield and fluorescence channels, respectively. To summarize, Active Contour is used in 2D bead velocity and 2D collagen density analyses. Canny Edge detection is used in 3D collagen density and 3D hole volume analyses.

## Supplementary Note 2: Simulations to validate our 3D collagen density measurements

To test the validity of our method, we simulate perfect-sphere spheroids of radius *R* (=100 pixel) with a uniform distribution of collagen beads around it. We plot normalized intensity of beads as a function of distance away from the center of the spheroid. We calculate the 2D collagen density by tracking bead distance on the same z-plane as the midsection of the simulated spheroid. By calculating the distance of every bead to the centroid, 3D resolution of collagen density is obtained. Here we show data of one of the simulated spheroids (Fig. S1C-D). In both 2D and 3D simulations, we observe a relatively uniform distribution of beads, which matches with the simulated distribution of the beads.

**Supplementary Figures**

**
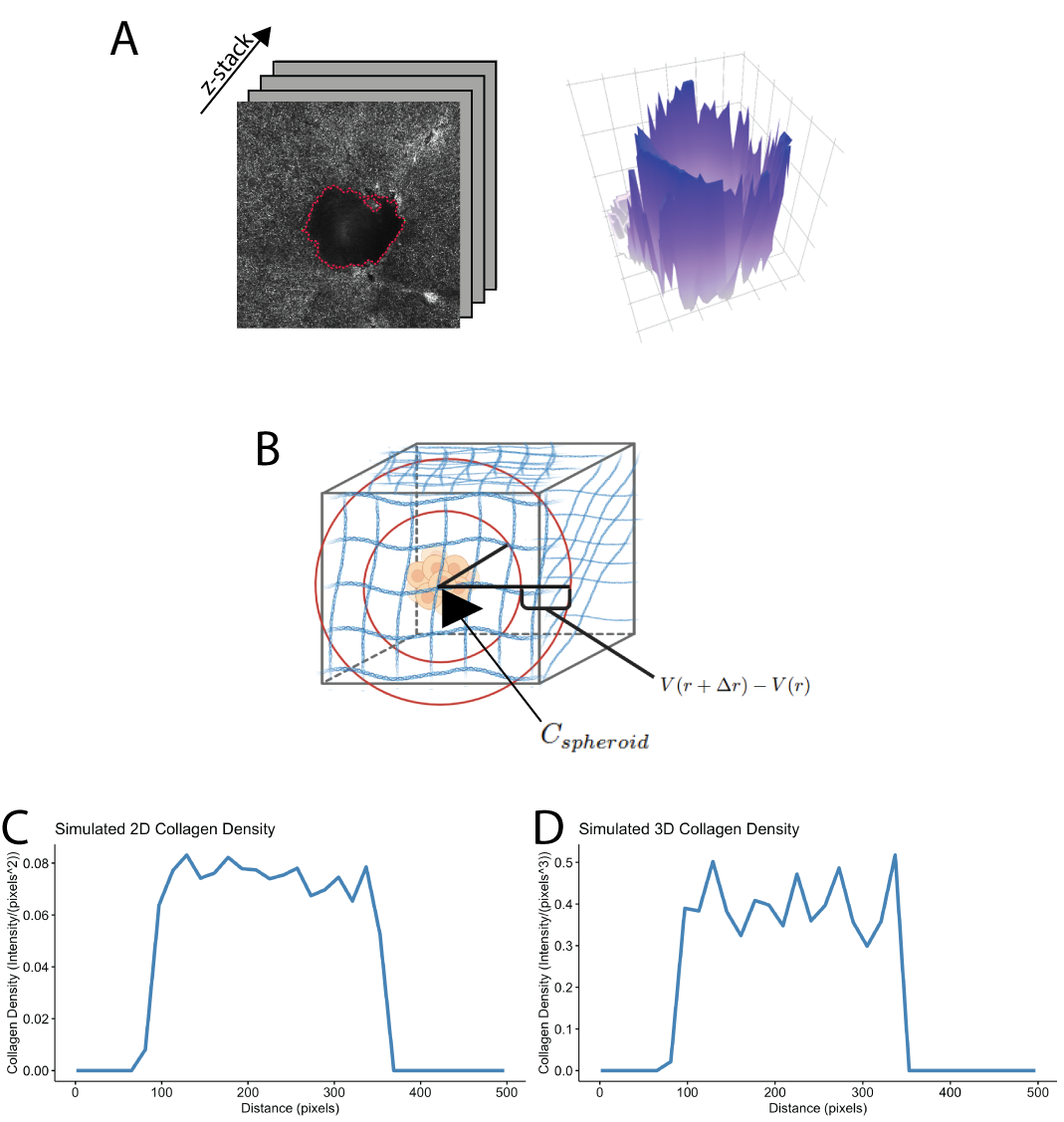
**

**Figure S1 | 3D hole volume and 3D collagen density measurement.** (A) A z-slice in a z-stack of images captured by reflectance microscopy showing the hole on day 6. The boundary of the hole, as detected by Canny Edge detection, is shown in red. Hole volume can be visualized as the 3D reconstruction on the right. (B) A schematic of 3D collagen density measurement. 3D collagen density is measured as the sum of the beads’ fluorescent intensities in the spherical shell divided by the volume of the spherical shell (V(r+Δr) - V(r)). Similarly, 2D collagen density is measured as the sum of the beads’ fluorescent intensities in the midsection’s ring divided by the area of the circular ring. The shell is centered at the spheroid’s centroid (C_spheroid_) and has a thickness of Δr=25 pixels. This figure is created with Biorender. (C) Simulated 2D collagen density as a function of the distance away from the centroid. (D) Simulated 3D collagen density as a function of the distance away from the centroid.


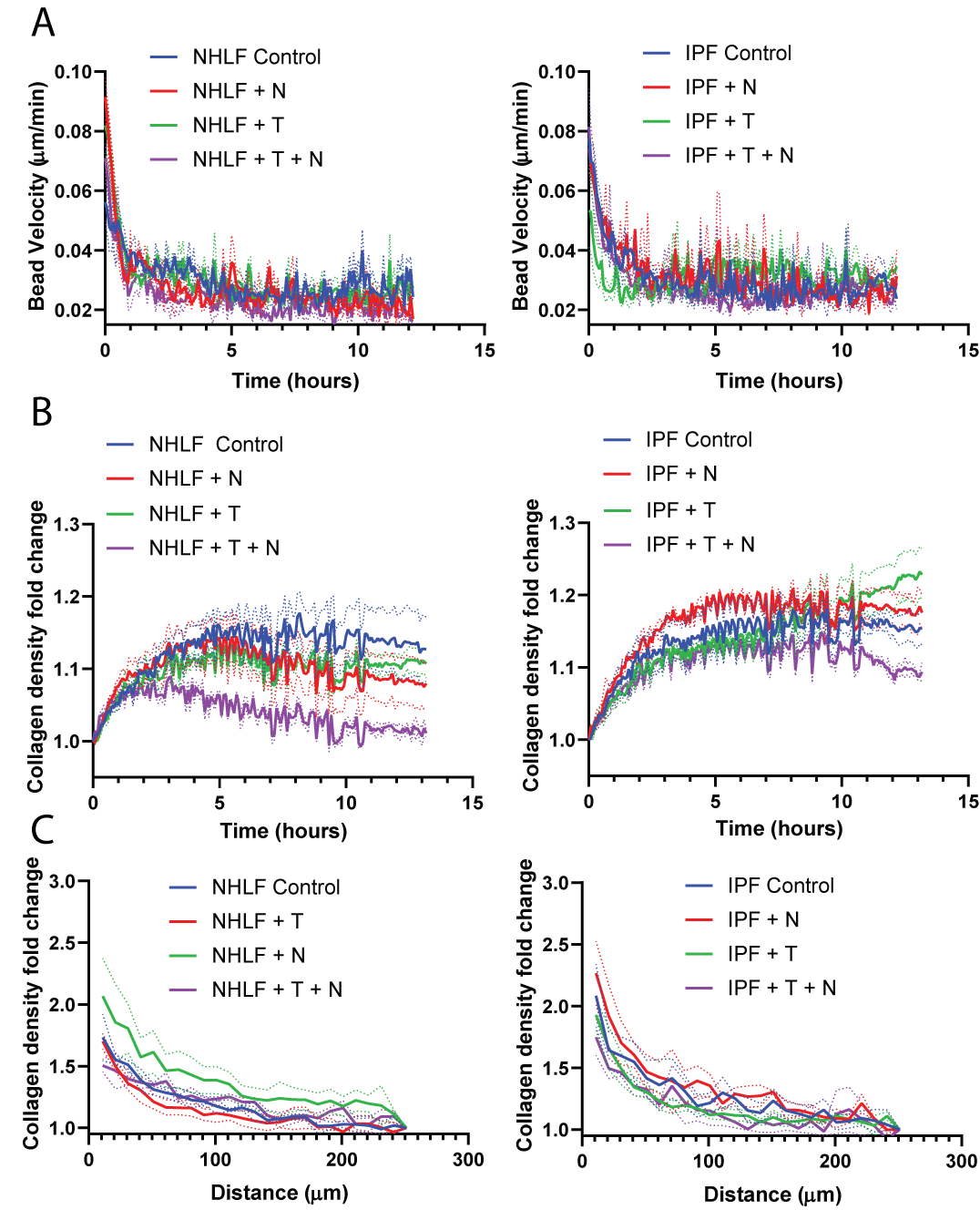


**Figure S2 | Nintedanib treatment 2D analysis.** A) Bead velocity near the spheroids is plotted over time for NHLF29800 and IPF29548 cell lines. B) 2D collagen density fold change is plotted over time for NHLF29800 and IPF29548 cell lines. C) 2D collagen density fold change over distance is plotted over the distance away from the spheroid for NHLF29800 and IPF29548 spheroids at hour 6. n≥2 spheroids for each condition. T stands for TGF-β. N stands for Nintedanib. The dotted lines represent the standard error of the mean (s.e.m.).

~~
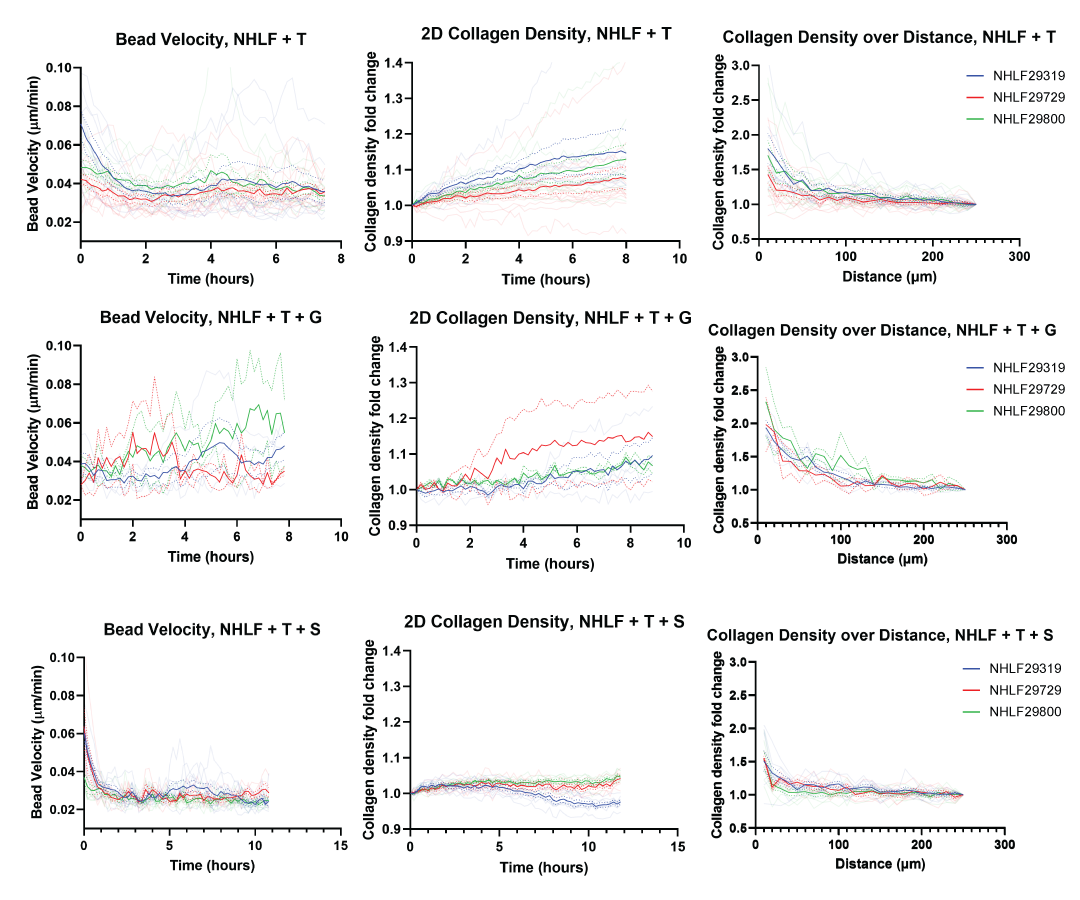
~~

**Figure S3 | GM6001 and SMIFH2 2D analysis for NHLF cell lines.** The plots show bead velocity (left column), intensity over time (middle column), and intensity over distance (right column) from 2D analysis. The solid lines are the means of each cell line. The dotted lines represent the standard error of the mean (s.e.m.). The transparent lines are data of individual spheroids. n≥2 spheroids for each condition. T stands for TGF-β. G stands for GM6001. S stands for SMIFH2.


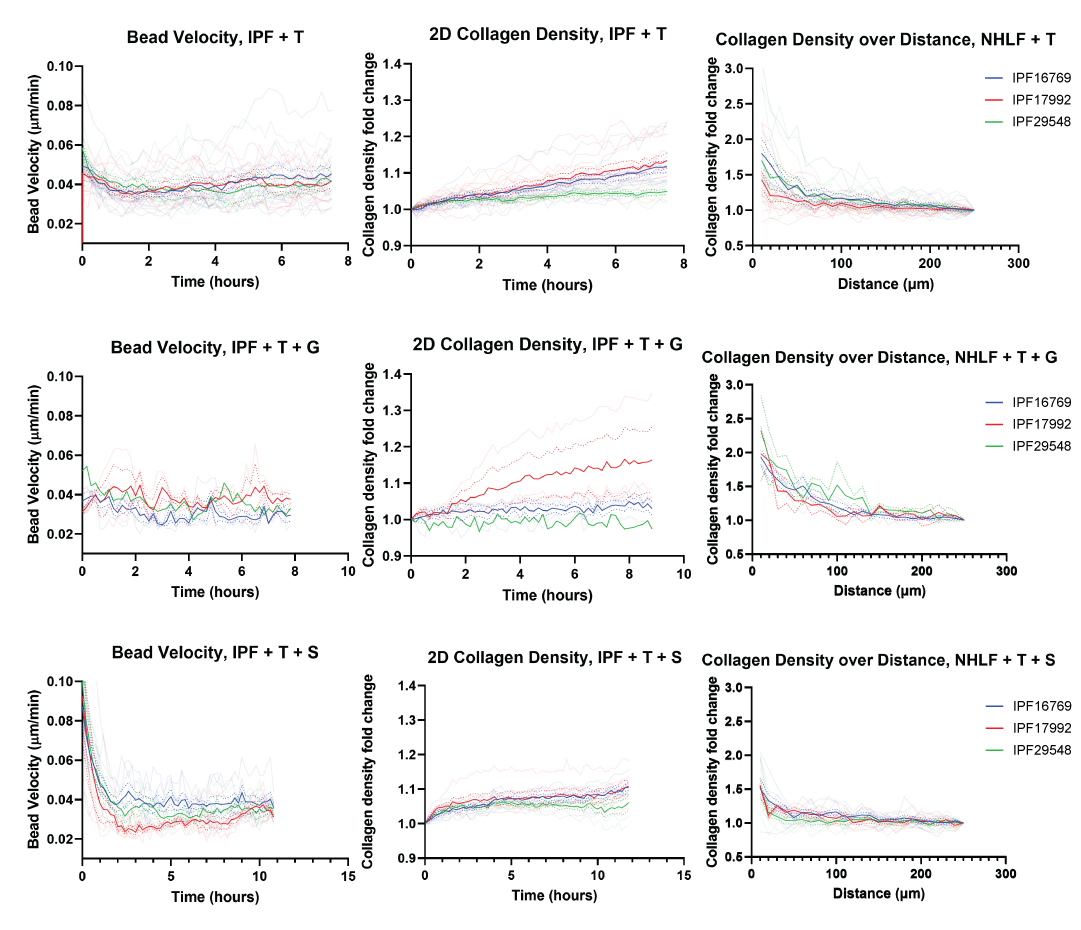


**Figure S4 | GM6001 and SMIFH2 2D analysis for IPF cell lines.** The plots show bead velocity (left column), intensity over time (middle column), and intensity over distance (right column) from 2D analysis. The solid lines are the means of each cell line. The dotted lines represent the standard error of the mean (s.e.m.). The transparent lines are data of individual spheroids. n≥2 spheroids for each condition. T stands for TGF-β. T stands for TGF-β. G stands for GM6001. S stands for SMIFH2.


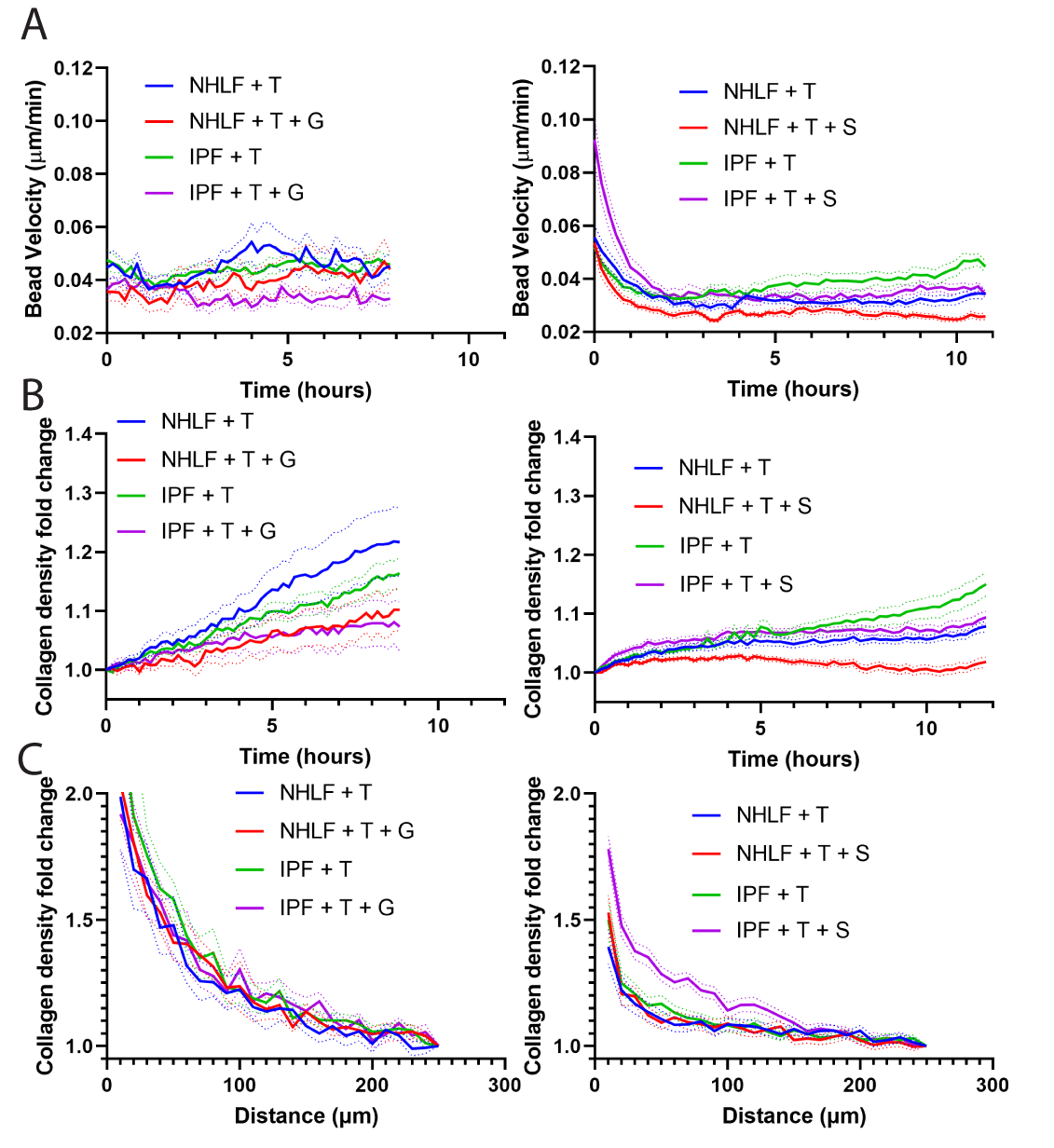


**Figure S5 | GM6001 and SMIFH2 2D analysis for NHLF and IPF cell lines when cell lines from different patients are pooled.** The plots show bead velocity (A), intensity over time (B), and intensity over distance (C) from 2D analyses. The dotted lines represent the standard error of the mean (s.e.m.). n≥2 spheroids for each condition. T stands for TGF-β. G stands for GM6001. S stands for SMIFH2.


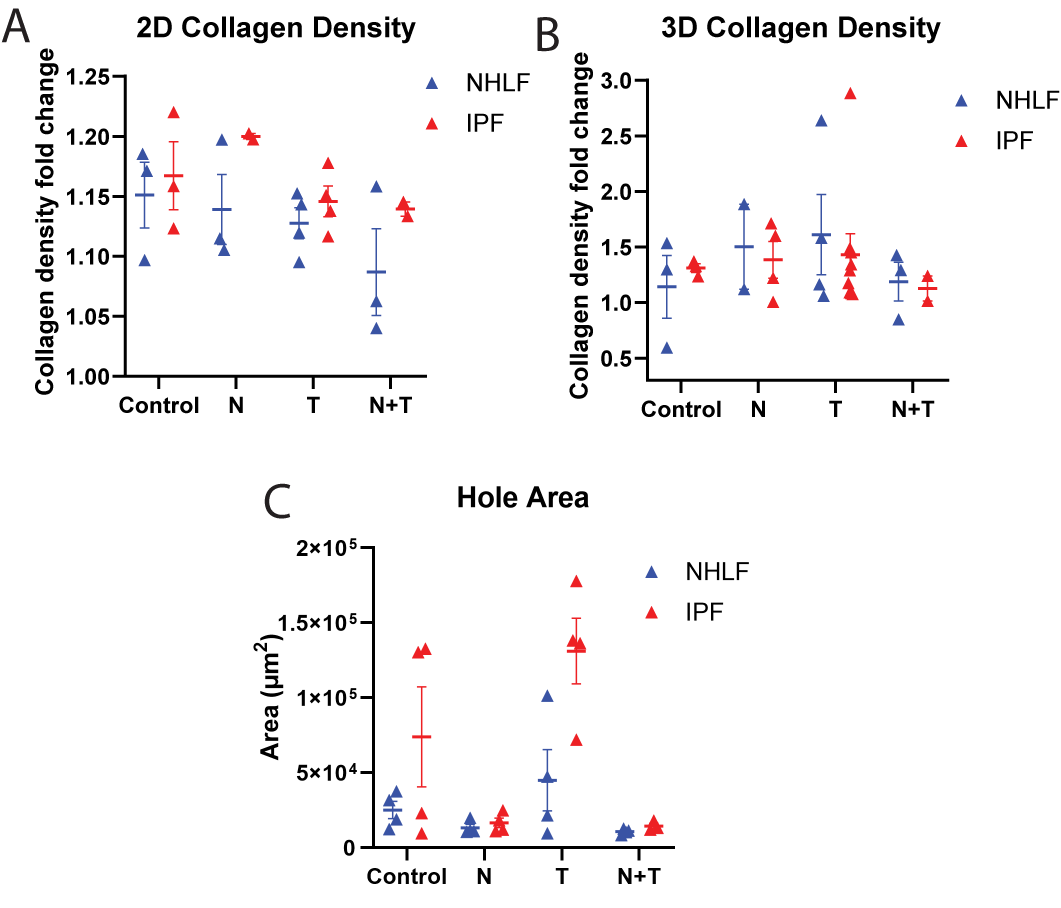


**Figure S6 | Comparison of NHLF29800 and IPF29548 spheroids treated with Nintedanib, TGF-β, TGF-β + Nintedanib.** (A) 2D collagen density fold change for IPF and NHLF spheroids at 6 hours after seeding. (B) 3D collagen density fold change for IPF spheroids at hour 6 after seeding. (C) Day 5 hole area for NHLF and IPF spheroids. T stands for TGF-β. N stands for Nintedanib.


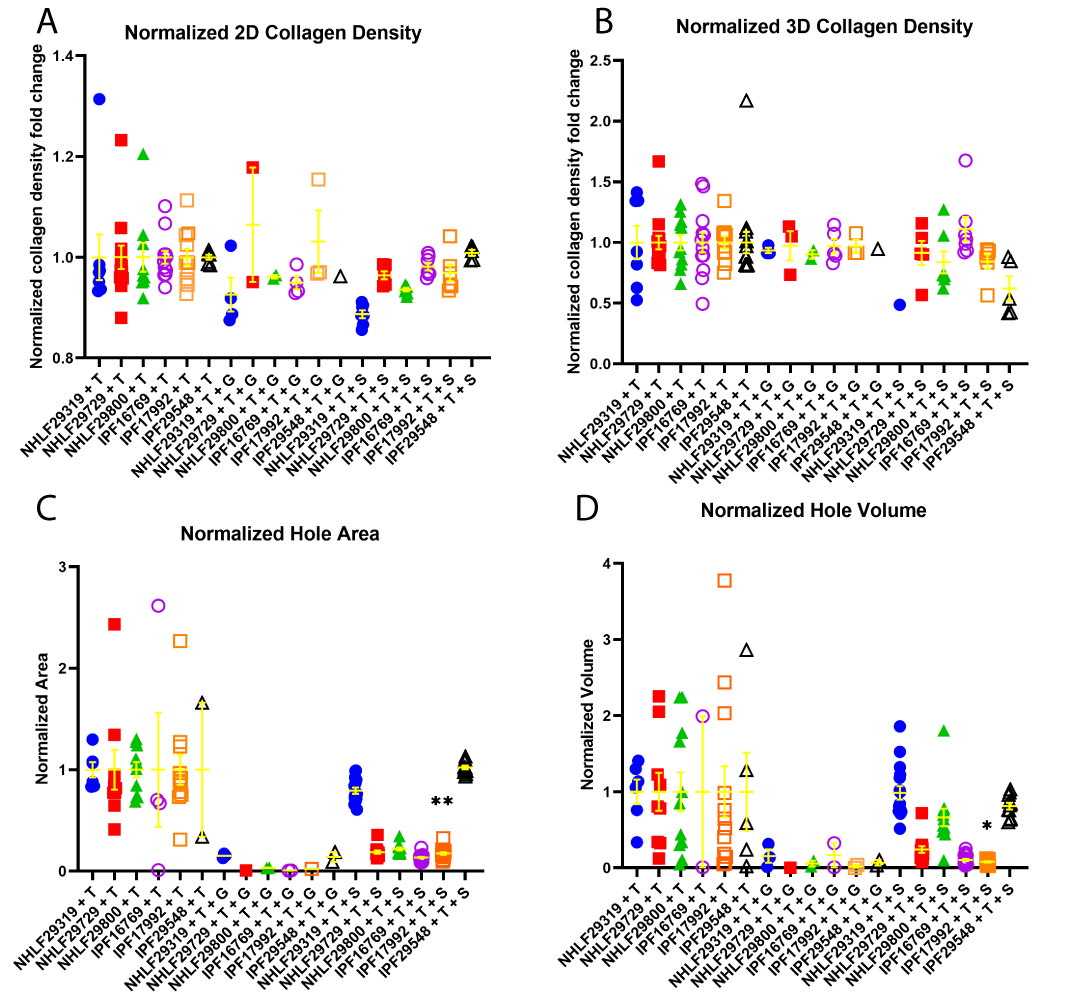


**Figure S7 | Comparison of individual NHLF and IPF cell lines under the GM6001 and SMIFH2 treatments normalized by the mean of the TGF-β treated group of the corresponding cell lines.** (A) 2D collagen density fold change at 6 hours after seeding normalized to each spheroid’s hour 0 collagen density and by the TGF-β only condition for each cell line for IPF and NHLF spheroids. Significance is assigned based on one-sample, two-tailed Wilcoxon ranked sign test with Benjamini-Hochberg correction. (B) 3D collagen density fold change at 6 hours after seeding normalized to each spheroid’s hour 0 collagen density and by the TGF-β only condition for each cell line for IPF and NHLF spheroids. Significance is assigned based on one-sample, two-tailed Wilcoxon ranked sign test with Benjamini-Hochberg correction. (C) Day 5 hole area normalized by TGF-β only condition for each cell line for NHLF and IPF spheroids. Significance for hole area differences is assigned based on one-way Kruskal Wallis test followed by Dunn post-hoc comparison tests. Asterisks are shown for the comparisons between each cell line with its own TGF-β only group. (D) Day 5 hole volume normalized by TGF-β only condition for each cell line for NHLF and IPF spheroids. Significance for hole volume differences is assigned based on one-way Kruskal Wallis test followed by Dunn post-hoc comparison tests. Asterisks are shown for the comparisons between each cell line with its own TGF-β only group. T stands for TGF-β. G stands for GM6001. S stands for SMIFH2.


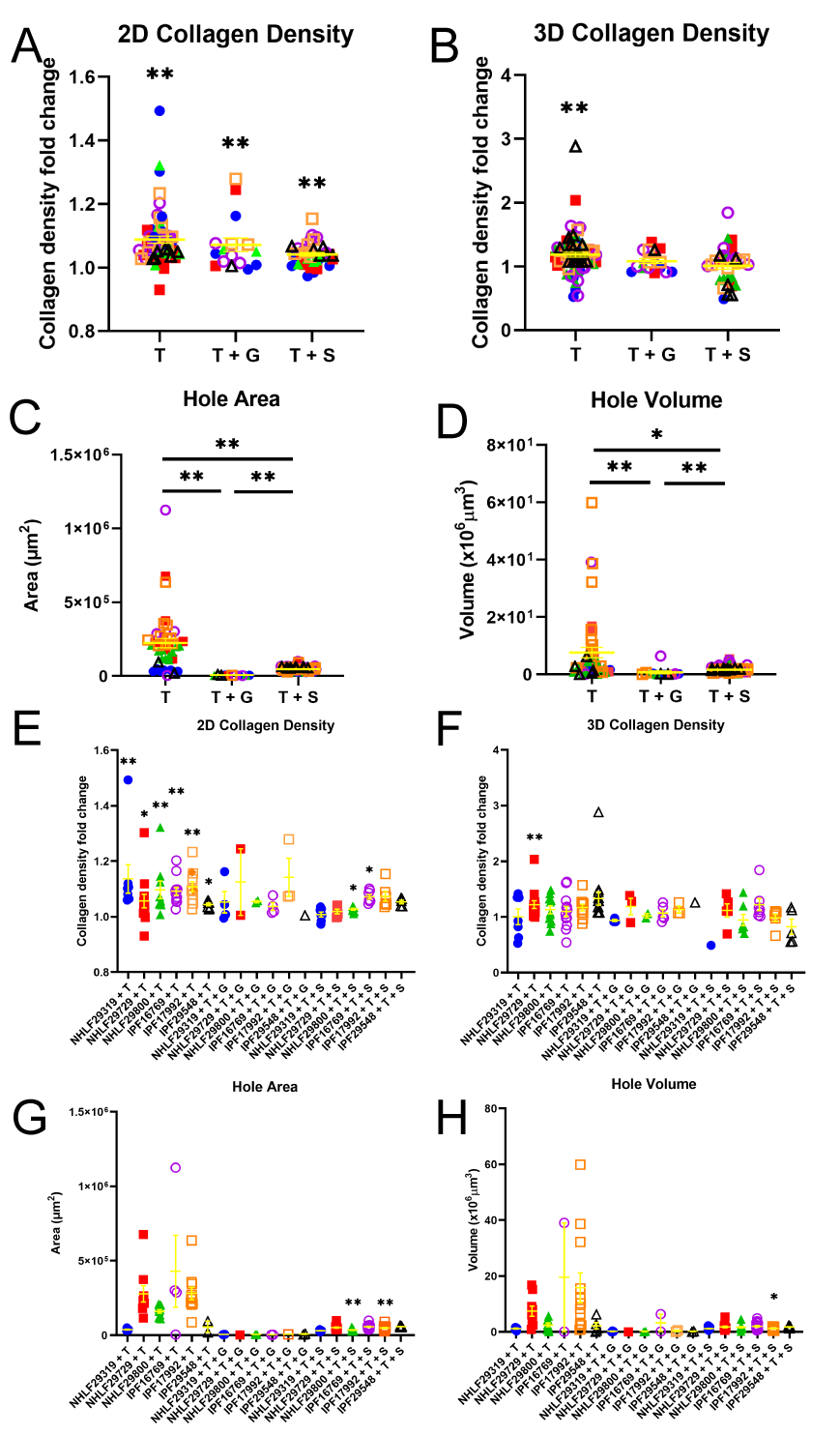


**Figure S8 | Analysis of GM6001 and SMIFH2 treatment conditions.** (A) 2D collagen density fold change for pooled IPF and NHLF spheroids at 6 hours after being cultured in collagen. Significance is assigned based on one-sample, two-tailed Wilcoxon ranked sign test with Benjamini-Hochberg correction. (B) 3D collagen density fold change for pooled IPF and NHLF spheroids at 6 hours after being cultured in collagen. Significance is assigned based on one-sample, two-tailed Wilcoxon ranked sign test with Benjamini-Hochberg correction. (C) Day 5 hole area for pooled NHLF and IPF spheroids. Significance for hole area differences is assigned based on one-way Kruskal Wallis test followed by Dunn post-hoc comparison tests. (D) Day 5 hole volume for pooled NHLF and IPF spheroids. Significance for hole volume differences is assigned based on one-way Kruskal Wallis test followed by Dunn post-hoc comparison tests. (E) 2D collagen density fold change for IPF and NHLF spheroids at 6 hours after being cultured in collagen. Significance is assigned based on one-sample, two-tailed Wilcoxon ranked sign test with Benjamini-Hochberg correction. (F) 3D collagen density fold change for IPF and NHLF spheroids at 6 hours after being cultured in collagen. Significance is assigned based on one-sample, two-tailed Wilcoxon ranked sign test with Benjamini-Hochberg correction. (G) Day 5 hole area for NHLF and IPF spheroids. Significance for hole area differences is assigned based on one-way Kruskal Wallis test followed by Dunn post-hoc comparison tests. Asterisks are shown for the comparisons between each cell line with its own TGF-β only group. (H) Day 5 hole volume for NHLF and IPF spheroids. Significance for hole volume differences is assigned based on one-way Kruskal Wallis test followed by Dunn post-hoc comparison tests. Asterisks are shown for the comparisons between each cell line with its own TGF-β only group. T stands for TGF-β. G stands for GM6001. S stands for SMIFH2.


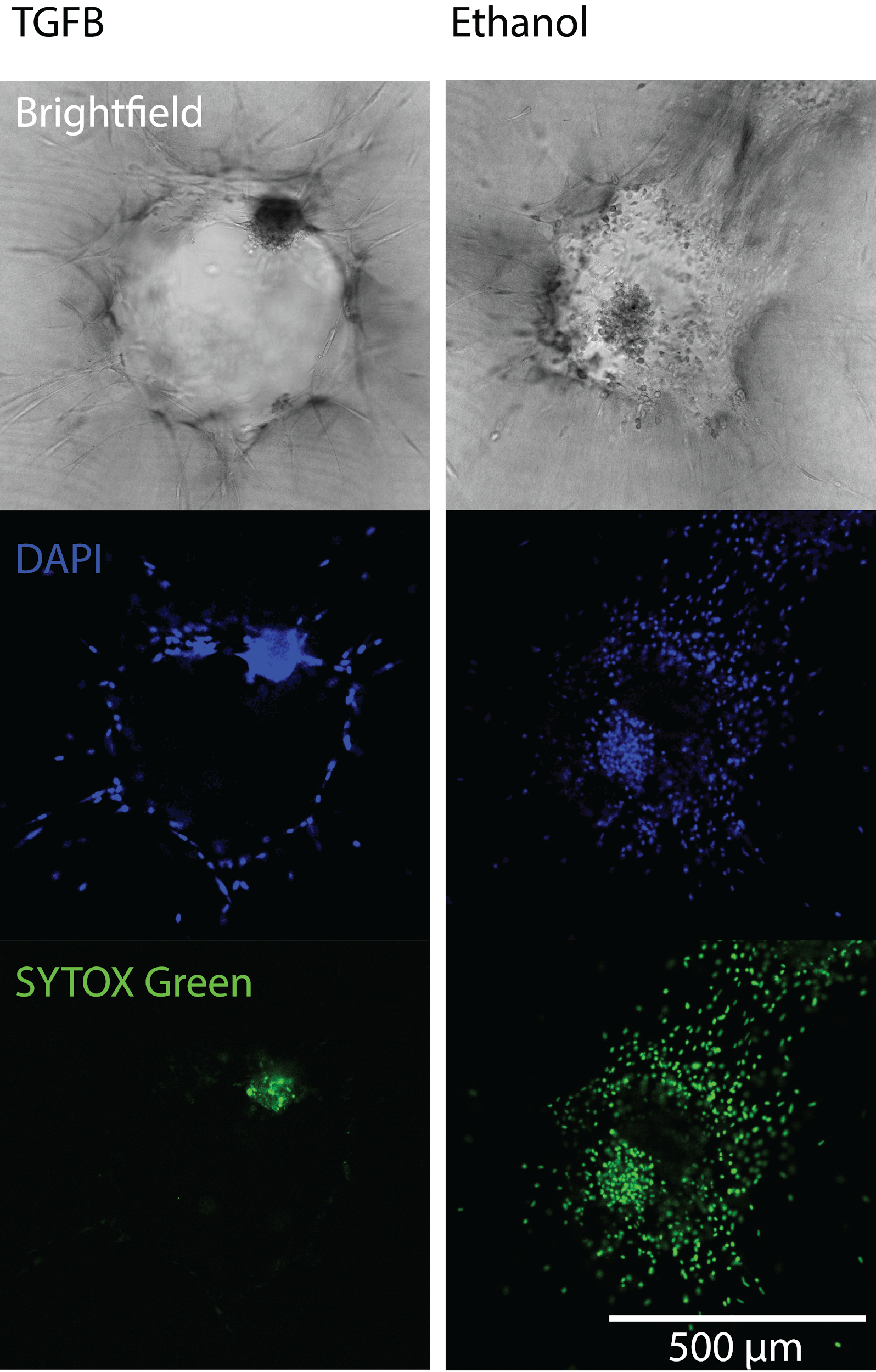


**Figure S9 | Viability fluorescence imaging of cells after 5 days of culture in collagen gels.** When spheroids cultured in collagen are exposed to 10 ng/ml TGF-β in cell culture media for 5 days, most cells remain alive, though there is a necrotic core (left column). As a control, the cells are also treated with 70% alcohol immediately before imaging on day 5 (right column). The 70% alcohol kills cells.

# References

1. [Kass, M., Witkin, A. & Terzopoulos, D. Snakes: Active contour models. *Int. J. Comput. Vis.* **1**, 321–331 (1988).](http://paperpile.com/b/z5cTKl/Xicy8)

2. [Canny, J. A computational approach to edge detection. *IEEE Trans. Pattern Anal. Mach. Intell.* **8**, 679–698 (1986).](http://paperpile.com/b/z5cTKl/WEDm0)
